# Intravenous infusion of small umbilical cord mesenchymal stem cells could enhance safety and delay retinal degeneration in RCS rats

**DOI:** 10.1186/s12886-021-02171-3

**Published:** 2022-02-11

**Authors:** Qingling Liang, Qiyou Li, Bangqi Ren, Zheng Qin Yin

**Affiliations:** 1 Southwest Hospital/Southwest Eye Hospital, Third Military Medical University (Amy Medical University), Chongqing, 400038 China; 2 Key Lab of Visual Damage and Regeneration & Restoration, Chongqing, 400038 China

**Keywords:** Umbilical cord mesenchymal stem cells, Intravenous infusion, Safety, Efficacy, Retinitis pigmentosa

## Abstract

**Background:**

Human umbilical cord mesenchymal stem cells (UCMSCs) transplantation is a promising therapy for the treatment of retinitis pigmentosa (RP). However, intravenously infused cells may be blocked in the lung, increasing the risk of vascular obstruction, which needs to be optimized to further improve safety and efficacy.

**Methods:**

We derived small UCMSCs (S-UCMSCs) from filtering UCMSCs with a 10-μm filter, and compared with UCMSCs by flow cytometry, directional differentiation culture and transcriptome sequencing. Then the S-UCMSCs and UCMSCs were intravenously infused in the Royal College Surgeons (RCS) rats to evaluate the safety and the efficacy.

**Results:**

The diameter of S-UCMSCs ranged from 5.568 to 17.231 μm, with an average diameter of 8.636 ± 2.256 μm, which was significantly smaller than that of UCMSCs. Flow cytometry, immunofluorescence and transcriptome sequencing demonstrated that the S-UCMSCs and UCMSCs were the same kind of MSCs, and the S-UCMSCs were more proliferative. After the S-UCMSCs and UCMSCs were intravenously infused into the Royal College of Surgeons (RCS) rats at a dose of 1 × 10^6^ cells/rat, the S-UCMSCs blocked in the lungs were significantly fewer and disappeared more quickly than UCMSCs. The b wave of the flash electroretinogram was improved at 7 d, and the retinal outer nuclear layer thickness was thicker at 7 d and 14 d. The expression level of inflammation was inhibited, and the expression level of neurotrophic factors was upregulated in the retina and serum after transplantation.

**Conclusions:**

S-UCMSCs intravenous infusion was safer than UCMSCs and could delay retinal degeneration and protect visual function in RCS rats, which may be a preferable therapeutic approach for RP.

**Supplementary Information:**

The online version contains supplementary material available at 10.1186/s12886-021-02171-3.

## Background

Retinitis pigmentosa (RP) is a common form of hereditary retinal degenerative disease characterized by photoreceptor and retinal pigmented epithelium (RPE) degeneration [1, 2]. A variety of treatments, such as neurotrophic factors, visual prosthesis devices and gene therapy [3–5], are trying to treat RP. Recently, the first US Food and Drug Administration (FDA)-approved gene therapy product, voretigene neparvovec-rzyl (Luxturna), was applied to the treatment of RPE65 gene-associated inherited retinal diseases, and promising results [6–8] have been encouraging for the treatment of RP.

Mesenchymal stem cells (MSCs) are multipotent stem cells characterized by self-renewal, multiple differentiation potentials, immunoregulation and neurotropic effects [9–11]. We have previously shown that bone marrow stem cells (BMSCs) could be induced toward functional retinal pigmented epithelium (RPE) cells or retinal neurons and secrete trophic factors under specific conditions in vitro [12, 13]. Therefore, MSC transplantation is considered to be a promising therapy for retinal degenerative diseases, such as wet age-related macular degeneration, Stargardt disease, and diabetic retinopathy [14, 15].

Intravenous infusion is a popular route for MSC transplantation in clinical trials [16, 17]. For retinal disease, it is noninvasive, low-cost, convenient and avoids the probable complications caused by vitreous cavity and subretinal transplantation, such as proliferation of fibrous tissue and traction retinal detachment [18–20]. And because of these advantages, intravenous infusion can be conducted repeatedly according to the patients’ condition, and it is more easily accepted and promoted by the public. Our previous clinical trials reported that intravenous infusion of MSCs could maintain or partially improve the visual function of advanced RP patients and nonproliferative diabetic retinopathy patients, and no side effects or complications were observed during these treatments [15, 21]. Furthermore, as RP is a kind of chronic progressive disease, along with the natural course of RP, the retinal microenvironment of patients with advanced RP is seriously damaged severely damaged, appearing ischemia and hypoxia, it is hardly to provide sufficient nutritional support or provide a good living environment for the intraocular transplanted cells. Therefore, intraocular transplantation may not be the most suitable treatment for advanced RP.

Umbilical cord mesenchymal stem cells (UCMSCs) are allogeneic MSCs derived from Wharton’s jelly with low immunogenicity and more biological advantages [22], and they can be obtained more safely and conveniently than BMSCs obtained from donor bone marrow that may carry pathogenic genes. It is reported that UCMSCs possess immunomodulatory properties including impairing the phagocytic and antigen-presenting capacity of monocytes/macrophages, inhibiting the maturation of dendritic cells and the generation properties of CD4+ T helper (Th)1 and Th17 cells, promoting the proliferation of Tregs and inhibiting the differentiation and antibody secretion of B cells [23–25]**.** And because of the immunosuppressive properties of UCMSCs, they have been used for the research of treatment of inflammatory disorders such as acute graft-versus-host disease, graft rejection in patients undergoing organ/cell transplantation, and autoimmune diseases etc., and no immune rejection was observed [26–28]. In addition, intravenous transplantation of UCMSCs can avoid immune rejection caused by retinal transplantation, so it may be the better choice for the treatment of RP.

However, some research has reported that the majority of intravascular infused MSCs might be blocked in the lungs and cause pulmonary vascular obstruction [29, 30], which increases the risk of transplantation. Although we ensured the safety of all subjects in our previous studies, with the increase in the number of subjects and number of administrations, there is also an increase in underlying risk of intravenous infusion of MSCs.

Therefore, further confirming the safety of intravenous infusion of UCMSCs and simultaneously maintaining their therapeutic role is our primary concern. In this study, we aimed to explore whether the intravenous infusion of small UCMSCs (S-UCMSCs) into Royal College of Surgeons (RCS) rats, a retinal degeneration animal model, could reduce the number of blocked cells in the lungs and simultaneously protect visual function.

## Methods

### Animal model

The Royal College of Surgeons (RCS) rat is the most classic and widely used animal model for studies of retinal degenerative disease [31, 32]. All RCS rats in this study are autosomal recessive rats with homozygous mutation of the tyrosine kinase Mertk gene, and they were provided by the Experimental Animal Center of the Army Medical University regardless of sex and raised in the Animal Care Center with free access to water and food. All animal experimental procedures were approved by the Institutional Review Board and the ethics committee of Southwest Hospital, Army Military Medical University, and conformed to the NIH guidelines for the ethical use of animals. All experiments involving human cells were carried out in accordance with the tenets of the Declaration of Helsinki, adhered to US Public Law 103–41, effective December 13, 2001.

### Grouping of UCMSCs

Human UCMSCs at passage 2 were obtained from Cyagen Biosciences, Suzhou, China, and all the cells met the following criteria. (1) Safety test criteria: asepsis, no endotoxin, no mycoplasma, and no hepatitis B or C; (2) quality test criteria: the positivity rate of CD11b, CD34 and CD45 was less than 5%, and the positivity rate of CD44, CD73, CD90 and CD105 was greater than 80%. The cell viability was not less than 90% [15]. A green fluorescence protein (GFP) lentiviral vector was used to transfect UCMSCs to track their distribution after intravenous infusion (Fig. S1). To obtain small UCMSCs, the cell suspension was filtered with a 10-μm filter (Sysmex, Japan), and the cells that passed through the pores were referred to as S-UCMSCs. The size of cells was evaluated by calculating the average diameter of 100 cells suspended in a culture dish by ImageJ software.

### Identification of MSC surface markers

Flow cytometry was used to examine surface markers. UCMSCs and S-UCMSCs were suspended in staining buffer, and PE-labeled antibodies against CD73, D90 and CD105 and FITC-labeled antibodies against CD34, CD45 and HLA-DR were added. After incubation in the dark for 30 min and washing with phosphate-buffered saline (PBS), cells were analyzed on a fluorescence-activated cell sorting (FACS) instrument and analyzed with FlowJo software.

### Differentiation ability of cells

UCMSCs and S-UCMSCs were cultured under specific conditions according to the manufacturer’s instructions (Cyagen, China) to induce differentiation to osteocytes, chondrocytes and adipocytes at 37 °C and 5% CO_2_ for 30 days. Then, the differentiation potential was evaluated by staining with oil red O for adipogenesis, Alizarin red for osteogenesis and Alcian blue for chondrogenesis.

### Cell cycle detection

Cells were prepared at a density of 1 × 10^6^ cells/tube. After fixation in 70% ice-cold ethanol at −20 °C for 1 h, 20 μl RNase A solution was added and incubated for 30 min at 37 °C. Then, 400 μl propidium iodide dyeing solution was added and incubated for 1 h at 4 °C. FACS was performed at an excitation wavelength of 488 nm within 24 h after staining, and data were analyzed with ModFit software.

### Transcriptome sequencing

Total RNA of S-UCMSCs and UCMSCs was extracted with TRIzol. cDNA libraries were sequenced on an Illumina sequencing platform by Genedenovo Biotechnology Co., Ltd. (Guangzhou, China). We analyzed the correlation between S-UCMSCs and UCMSCs based on the gene expression profile.

### Intravenous administration

S-UCMSCs and UCMSCs were suspended at a density of 2 × 10^6^ cells/ml in 0.5 ml balanced salt solution (BSS) and administered via the tail vein using a 30G needle to RCS rats (30 days old). RCS rats that received PBS or untreated rats served as the control groups. Three RCS rats in both the S-UCMSC group and UCMSC group were observed to evaluate the cells blocked in the lungs at 1, 12, 24, 48 and 72 h after transplantation, and 4 RCS rats in each experimental group were observed until 7, 14 and 28 d after transplantation to explore the efficacy of this treatment.

### Euthanize animals

According to the experimental progress, at each observation time, the RCS rats of corresponding groups were placed in the carbon dioxide (CO_2_) chamber and euthanized within 5 min as the CO_2_ filled the chamber at a rate of 10 to 30% of the box volume per minute.

### Counting GFP-positive cells in the lung

The whole lung was immediately removed when RCS rats were sacrificed at 1, 12, 24, 48 and 72 h after transplantation to make frozen sections, and DAPI staining was performed. To analyze the number of cells blocked in the lung, in the 5 lobes, 3 high power fields (× 400) were randomly selected from every lobe of each lung and observed by confocal laser scanning microscopy. The number of nuclei covered with green fluorescence was statistically analyzed.

### Stretched preparation and frozen sectioning of the retina

Eyes were obtained for retinal preparation at 1, 12, 24, 48 and 72 h after transplantation. After fixation in 4% PFA for 3 h, the whole retina was removed and placed on glass slides. At 7, 14 and 28 days after transplantation, the eyes were sectioned at 10 μm through the optic nerve along the vertical meridian within a radius of 1 mm to make frozen sections of the retina. To evaluate the degeneration of photoreceptors, we randomly selected 10 tissue samples and measured the outer nuclear layer (ONL) thickness with ImageJ software.

### Electrophysiological examination

Flash electroretinogram (FERG) examination was performed with a visual electrophysiology instrument (MAYO, Japan) before animals were sacrificed and at 7, 14 and 28 days after transplantation. The amplitude and latent time of the b wave were recorded at an intensity of 3.0 (P) d.s/m^2^.

### ELISA assay

When the RCS rats were sacrificed at 7, 14 and 28 days after transplantation, the RPE-Bruch’s membrane choriocapillaris complexes were removed carefully and homogenized, and the supernatant was collected after centrifugation. Blood samples of the RCS rats were obtained from the left ventricle, and serum was collected after centrifugation.

After the samples were collected, ELISAs were performed according to the manufacturer’s instructions (Cusabio, China) for interleukin-6 (IL-6), interleukin-10 (IL-10), brain-derived neurotrophic factor (BDNF), ciliary neurotrophic factor (CNTF), basic fibroblast growth factor (bFGF) and hepatocyte growth factor (HGF).

### Statistical analysis

Descriptive statistics were performed using GraphPad Prism 6.0. Data are presented as the mean ± SD. Student’s t-test was used for two groups, while two-way ANOVA was used for multiple data analysis and statistical analysis. A value of *P* < 0.05 was considered statistically significant.

## Results

After intravenous infusion of S-UCMSCs and UCMSCs into RCS rats through the tail vein, no abnormal reactions in daily dietary activities were observed, and no immune rejection or tumorigenesis in the retina were found by stretch preparation or frozen section throughout the 28 days.

### Biological characteristics of S-UCMSCs and UCMSCs

**Fig. 1:**
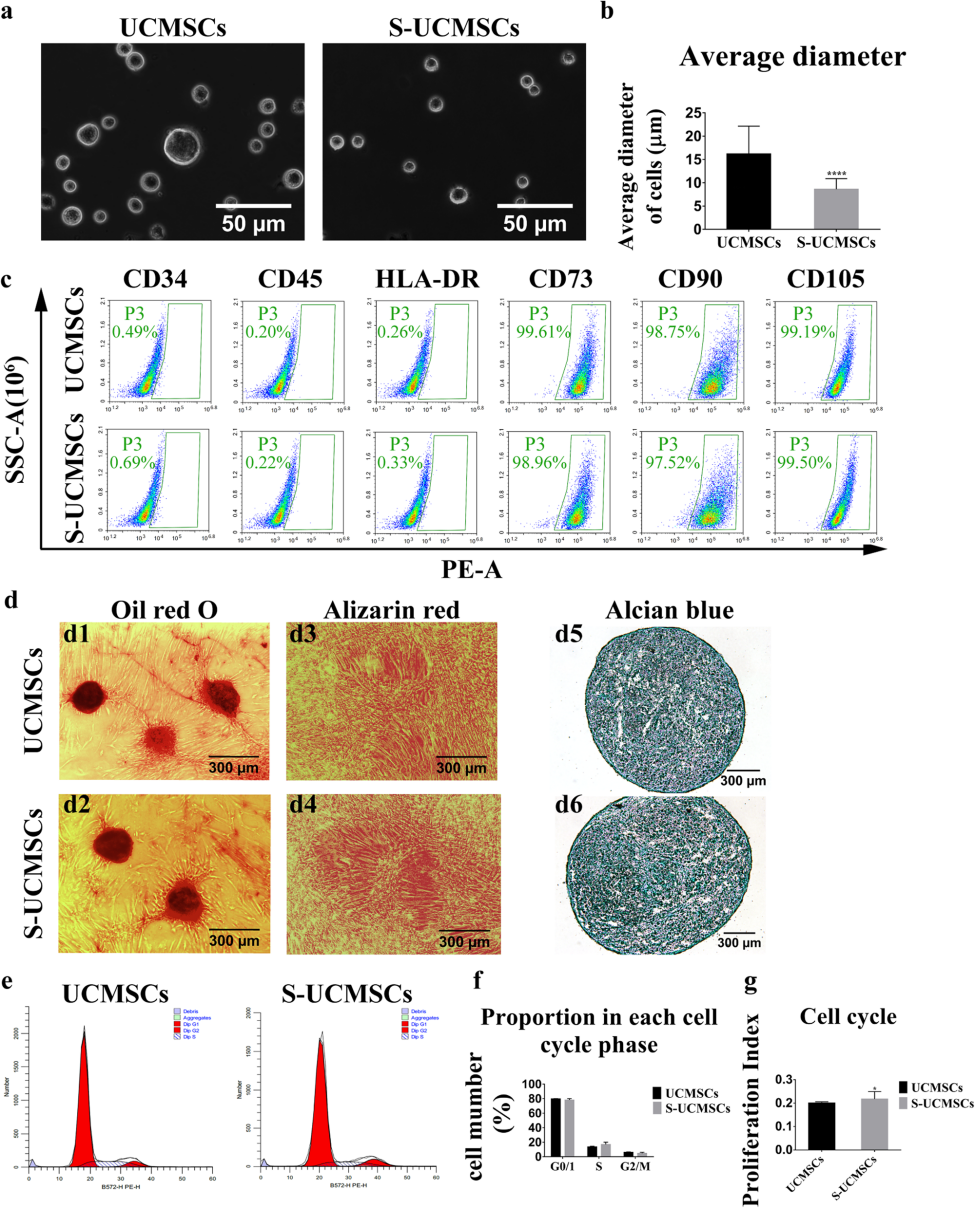
Comparison of the morphology and biological characterization of S-UCMSCs and UCMSCs. **a** In suspension culture, S-UCMSCs were significantly smaller than UCMSCs. **b** Statistical analysis of the average diameter of S-UCMSCs and UCMSCs (n = 100). **c** Flow cytometry showed that surface antigens of both S-UCMSCs and UCMSCs were positive for CD73, D90 and CD105 and negative for CD34, CD45 and HLA-DR. **d** Both S-UCMSCs and UCMSCs could be induced to differentiate into adipocytes, osteocytes and chondrocytes, which was confirmed by oil red O, Alizarin red and Alcian blue staining. **e** Representative cell cycle histograms from FACS. **f** The proportion of cells in each cell cycle phase (n = 3). **g** Statistical analysis showed that the PI of S-UCMSCs was higher than that of UCMSCs (n = 3). *P < 0.05, ****P < 0.001 versus UCMSCs. Scale bars: 20 μm (a), 100 μm (D1 and D2), 200 μm (D3 and D4), and 50 μm (D5 and D6)

The size of cultured UCMSCs at passage 5 in suspension conditions ranged from 6.364 to 39.395 μm, with an average diameter of 16.205 ± 5.947 μm (Fig. 1a). The size of S-UCMSCs that passed through 10-μm nylon filters ranged from 5.568 to 17.231 μm, with an average diameter of 8.636 ± 2.256 μm, which was significantly smaller than that of UCMSCs (Fig. 1b). Surface antigen detection showed that UCMSCs and S-UCMSCs shared a similar surface antigen pattern: both were highly positive for CD73, D90 and CD105 and negative for CD34, CD45 and HLA-DR (Fig. 1c), which meets the minimal criteria for defining MSCs [33].

After 30 days of adipogenic differentiation, the morphology and quantity of oil red O-positive lipid vacuoles in UCMSCs were very similar to those in S-UCMSCs. Both kinds of cells could be induced to differentiate into cells with similar mineralized nodules (stained with Alizarin red) in osteogenic differentiation assays and with proteoglycan pellets (stained with Alcian blue) in chondrogenic differentiation assays (Fig. 1d).

The proliferative capacity of UCMSCs was assessed by the proliferation index (PI = (S+ G2/M)/(G0/1 + S + G2/M). The percentage of cells in each cell cycle phase revealed that more S-UCMSCs than UCMSCs were in S phase, but fewer were in G0/1 and G2/M phases (Fig. 1e and f). The PI of S-UCMSCs was significantly higher than that of UCMSCs (Fig. 1g).

**Fig. 2:**
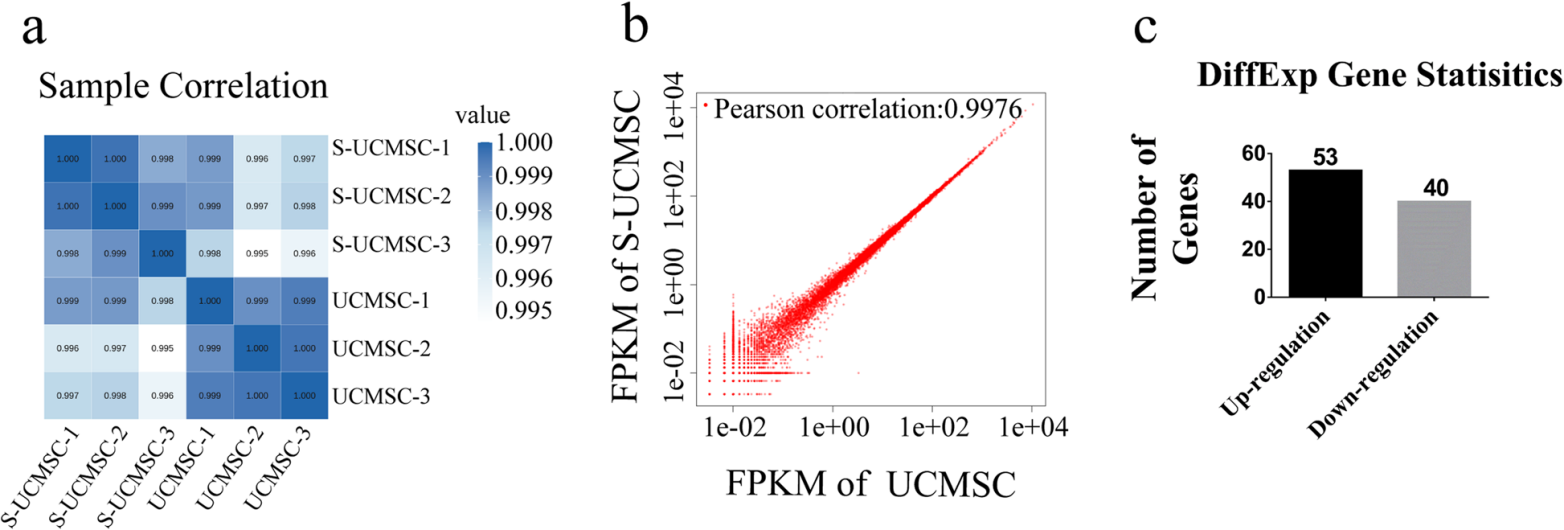
Correlation analysis of gene expression by transcriptome sequencing. **a** The correlation heat map demonstrated that the gene expression profiles of S-UCMSCs and UCMSCs were very similar. **b** The scatter plot showed that the Pearson correlation coefficient was 0.9976 between UCMSCs and S-UCMSCs. **c** Statistical analysis of differentially expressed genes showed that 53 genes were upregulated and 40 genes were downregulated in UCMSCs versus S-UCMSCs

Transcriptome sequencing showed that UCMSCs and S-UCMSCs were closely related in the correlation heat map (Fig. 2a), and the Pearson correlation coefficient of the two groups of cells was 0.9976 (Fig. 2b), which demonstrated that S-UCMSCs and UCMSCs were the same kind of cells with similar gene expression profiles, despite the upregulation of 53 genes and downregulation of 40 genes (Fig. 2c).

### Fewer S-UCMSCs than UCMSCs were blocked in the lungs of RCS rats

**Fig. 3:**
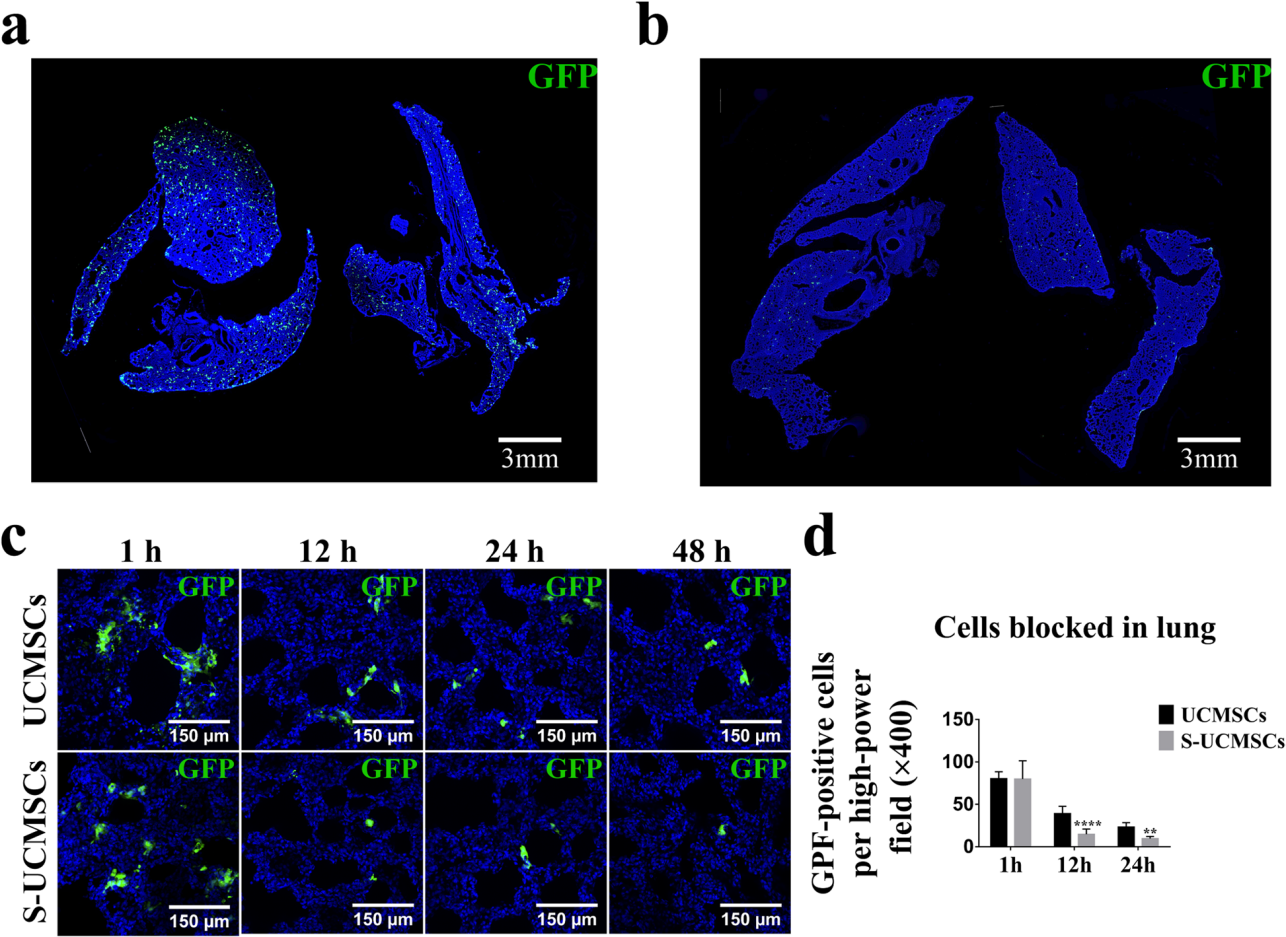
Comparison of the number of blocked cells in the lungs after transplantation. Representative micrographs of the whole lung fields of the S-UCMSC group at 1 h (**a**) and 24 h (**b**) showed that the number of blocked S-UCMSCs in the lung decreased rapidly after transplantation. **c** There were many GFP-positive cells in both the S-UCMSC and UCMSC groups at 1 h after transplantation, and there were significantly fewer S-UCMSCs blocked in the lungs than UCMSCs at 12 h and 24 h. **d** Statistical analysis of GFP-positive cells blocked in the lungs after transplantation (n = 3). **P < 0.01; ****P < 0.001 versus UCMSCs. Scale bar: 3 mm (**a** and **b**) and 100 μm (**c**)

S-UCMSCs and UCMSCs were intravenously infused into RCS rats through the tail vein, and the lungs were collected at 1, 12, 24, 48 and 72 h after transplantation. As shown in Fig. 3, 1 h after transplantation, a large number of GFP-positive cells were blocked in the lungs in both groups. Then, the number of S-UCMSCs blocked in the lung quickly decreased (Fig. 3a and b), and significantly fewer S-UCMSCs than UCMSCs were in the lungs at 12 h and 24 h after transplantation (Fig. 3c). Forty-eight hours later, S-UCMSCs had almost disappeared, with too few cells retained in the lung for statistical analysis, while residual UCMSCs could still be observed (Fig. S2). Furthermore, no GPF-positive cells were observed in the lungs at 72 h after transplantation.

### S-UCMSCs and UCMSCs have similar protective effects in RCS rats

**Fig. 4:**
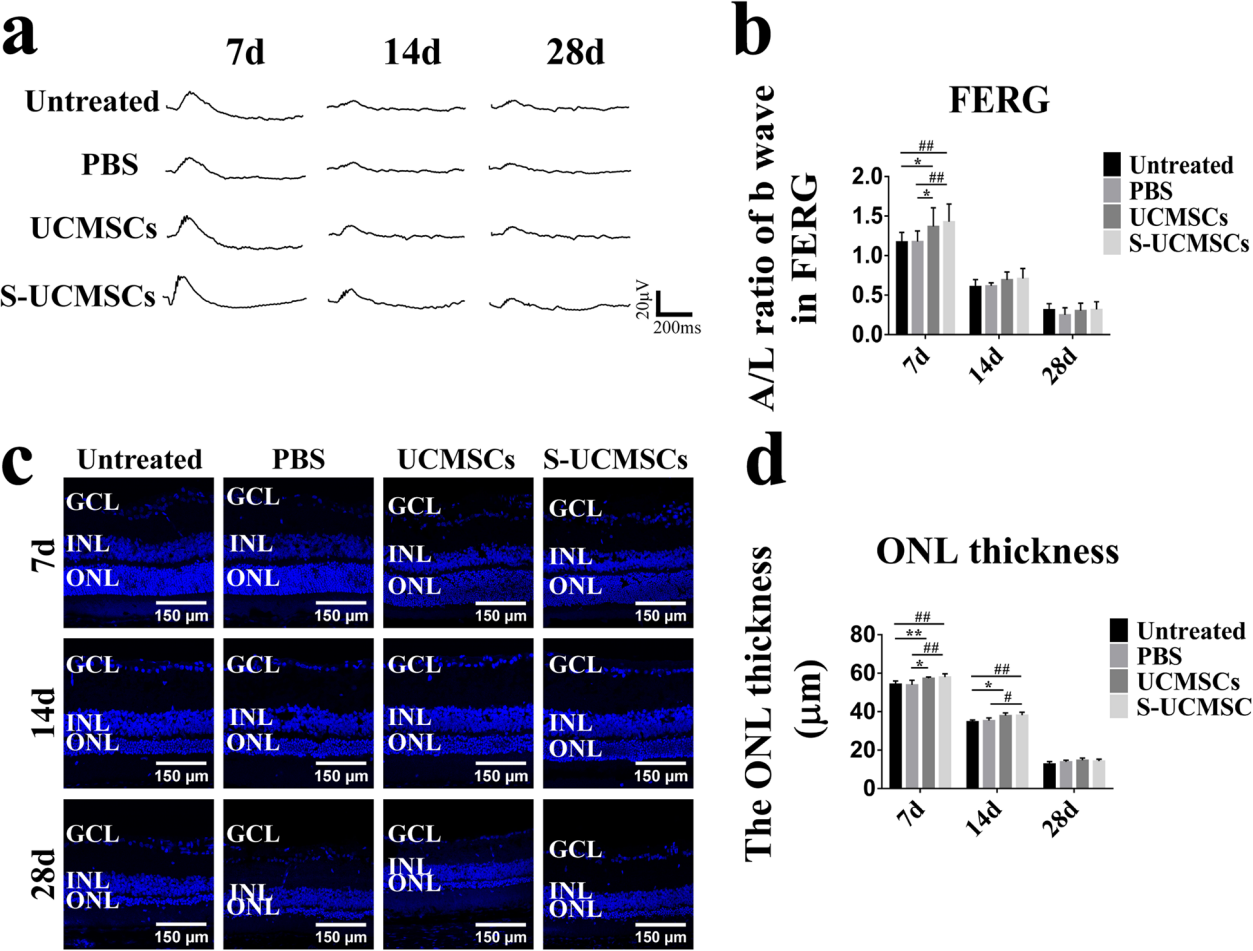
The effectiveness of S-UCMSCs and UCMSCs in RCS rats. **a** Representative waveforms of FERG b waves after transplantation. **b** Statistical analysis of the amplitude/latency (A/L) ratio of FERG b waves showed that the amplitude/latency of the S-UCMSC and UCMSC groups was significantly higher than that of the untreated and PBS groups (n = 8). **c** Representative micrographs of frozen sections of retinas stained with DAPI at 7 d, 14 d and 28 d after transplantation. **d** Statistical analysis of the ONL thickness (n = 4). *P < 0.05; **P < 0.01 versus UCMSCs; ^*#*^*P* < 0.05; ^##^*P* < 0.01 versus S-UCMSCs; Scale bar: 100 μm

Flash Electroretinogram (FERG) tests showed that the average amplitude/latency (A/L) ratio of the b wave of the UCMSC and S-UCMSC groups was higher than that of the PBS and untreated groups at 7 and 14 days after intravenous infusion (Fig. 4a), and the difference at 7 days was statistically significant (Fig. 4b). With the progression of retinal degeneration, the amplitude of the b wave was hardly observed at 28 days in all groups.

The ONL thickness of the S-UCMSC and UCMSC groups was significantly thicker than that of the PBS and untreated groups at 7 days and 14 days after transplantation (Fig. 4c and d). With the natural course of retinal degeneration, only approximately 3–5 layers remained in the ONL in all groups 28 days after transplantation.

**Fig. 5:**
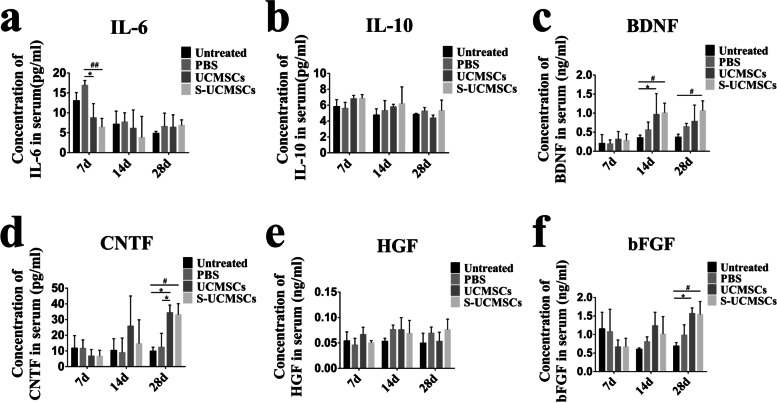
Statistical analysis of the level of cytokines in the RPE-Bruch’s membrane choriocapillaris complexes. **a** ELISA showed that the level of IL-6 significantly decreased at 14 d and 28 d, (**b**) and that of IL-10 significantly increased until 28 days after UCMSC and S-UCMSC intravenous transplantation. **c** The levels of BDNF were significantly increased at 14 d after transplantation and those of bFGF were increased at 7 d and 14 d (**e**). **d** and **f** The levels of CNTF and HGF were significantly continuously upregulated until 28 days after intravenous infusion (n = 3)**.** *P < 0.05; **P < 0.01; ***P < 0.005; ****P < 0.001 versus UCMSCs; ^*#*^*P* < 0.05; ^##^*P* < 0.01, ^###^*P* < 0.005; ^####^*P* < 0.001 versus S-UCMSCs

The mechanism of UCMSC intravenous transplantation is mainly attributed to anti-inflammatory and neurotrophic effects [34, 35]. The ELISA showed that the expression level of IL-6 in the RPE-Bruch’s membrane choriocapillaris complexes of the RCS rats’ retina in the S-UCMSC and UCMSC groups was significantly lower and the level of IL-10 was much higher than those in the PBS and untreated groups after transplantation (Fig. 5a and b). Meanwhile, the levels of BDNF in the two transplanted groups were significantly higher at 14 d and that of bFGF was upregulated at 7 d and 14 d compared with the two control groups, and the levels of CTNF and HGF were significantly continuously upregulated until 28 days after transplantation (Fig. 5c-f).

**Fig. 6:**
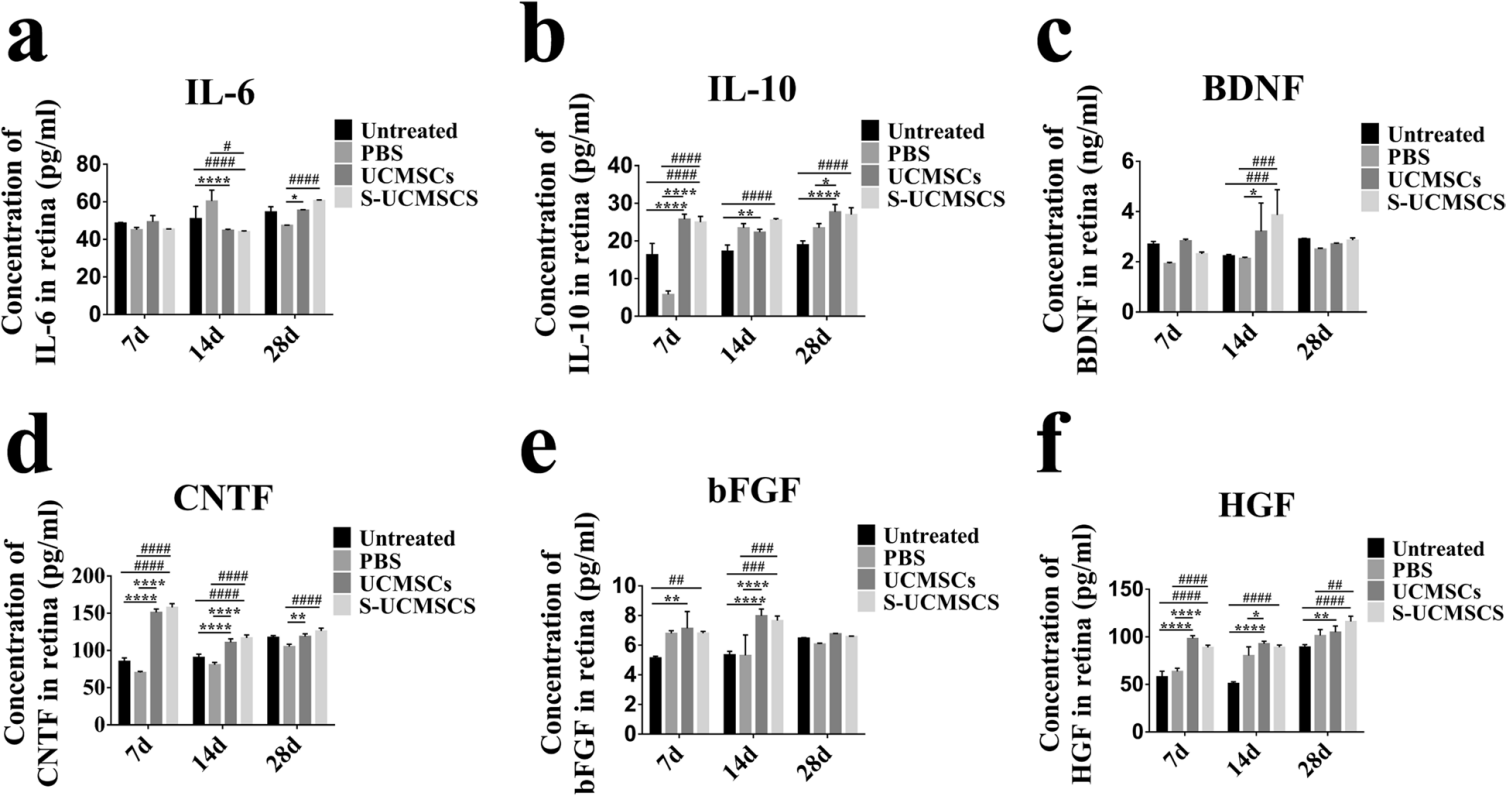
Statistical analysis of the expression level of cytokines in the serum of RCS rats. **a** and **b** ELISA showed that the level of IL-6 in serum significantly decreased at 7 d after transplantation (n = 3) and that the level of IL-10 displayed an increasing trend. **c**-**f** The levels of BDNF, CTNF and bFGF were significantly upregulated after cell infusion at 14 d and 28 d after transplantation and that of HGF was not significantly changed (n = 3)**.** *P < 0.05; **P < 0.01 versus UCMSCs; ^*#*^*P* < 0.05; ^##^*P* < 0.01 versus S-UCMSCs

Furthermore, we found that the level of IL-6 in the serum of RCS rats in the S-UCMSC and UCMSC groups was lower than that in the PBS and untreated groups at 7 days, and the difference with the PBS group was significant (Fig. 6a). The level of IL-10 tended to increase at 7 and 14 days after transplantation (Fig. 6b). Meanwhile, the expression levels of BDNF, CNTF and bFGF in the two transplanted groups were significantly upregulated at 14 d or 28 d after treatment (Fig. 6c-f). In addition, compared with UCMSCs, S-UCMSCs displayed no significant advantage in regard to the therapeutic effect.

## Discussion

UCMSCs are an ideal cell source for stem cell transplantation [36]. Compared with MSCs derived from the bone marrow, adipose tissue and other sources, UCMSCs avoid ethical controversy, and they are derived through noninvasive means without pathogenic genes carried by the donor [37, 38]. Studies have reported that UCMSC transplantation can be safely applied to a variety of diseases and has shown some positive effects [39–42]. However, evidence has shown that the majority of intravenously delivered MSCs are blocked inside the lungs, which might cause pulmonary vascular obstructions to increase the risk of transplantation [43, 44]. Therefore, it is necessary to optimize MSC intravenous infusion to further improve safety. Although the mechanisms of vascular obstruction are poorly understood, the size of cells has been assumed to be the key factor [45]. We obtained S-UCMSCs by filtering UCMSCs with a 10-μm nylon filter. Compared with UCMSCs, S-UCMSCs were more proliferative. Intravenously infused S-UCMSCs were less blocked in the lung and disappeared faster than UCMSCs. Moreover, S-UCMSCs provided retinal protection similar to that of UCMSCs.

To our knowledge, no studies have explored the safety and efficacy of the intravenous infusion of S-UCMSCs in RCS rats. We previously conducted phase II clinical trials on the intravenous infusion of MSCs in RP and diabetic retinopathy patients, and safety and promising therapeutic effects were observed. In addition, the safety of intravenous infusion has also been proven by meta-analysis [16] and other clinical studies [46, 47]. Therefore, intravenous infusion of UCMSCs is regarded as a safe approach based on the large number of previous studies and our clinical trial. However, no drugs or approaches can guarantee absolutely safe even if they have been widely applied to clinical treatment, which is the case for intravenous infusion. Unfortunately, as pulmonary capillaries with an average diameter of 6 ~ 9 μm are too small for UCMSCs with an average diameter of 16.205 ± 5.947 μm to pass through, they are considered to be the major obstacle for intravascular infused cells to migrate to other tissues [44, 48]. Therefore, to make the cells that infused intravenously pass through the pulmonary capillaries as many as possible, a 10 μm nylon filter was selected, which is the closest available size to the capillary diameter to obtain the S-UCMSCs. In this study, we clearly found that significantly fewer S-UCMSCs, with an average diameter of 8.636 ± 2.256 μm, were blocked in the lung than UCMSCs at 12 h and 24 h after transplantation, which might reduce the risk of vascular embolism. Consistent with our observations, Zanetti et al. [49] revealed that smaller BMSCs, with a diameter of 15.1 ± 0.9 μm, had an increased capacity to pass through the pulmonary microcirculation compared with traditional MSCs, and Ge et al. [50] also pointed out that the size of intrainternal artery-injected MSCs is a significant cause of vascular obstructions and stroke. Therefore, small MSCs may be a key factor in improving the safety of intravenous transplantation. Moreover, similar to other experiments [45, 51], we observed that S-UCMSCs displayed a more active proliferative state, and this superiority might play an important role in their rapid passage through the lung and therapeutic function.

The effectiveness of MSCs for the treatment of retinal degeneration has been proven in our previous studies [19, 52]. Then, did the difference in UCMSC size influence their function? In the present study, we observed that S-UCMSCs could protect visual function and photoreceptors by improving the FERG b wave and reducing the loss of ONL cells, which indicated that S-UCMSCs could delay retinal degeneration as effectively as UCMSCs. However, interestingly, neither S-UCMSCs nor UCMSCs homing to the retina were observed after intravenous infusion (Fig. S3). This might largely be attributed to obstruction of the lungs, while the blood retinal barrier might also be one of the reasons. Indeed, the homing of MSCs has always been discussed. Some studies demonstrated that intravenously administered MSCs could migrate to the sites of injury mediated by chemokines and the recruitment of the injured tissue [53–55]. However, Eggenhofer et al. [29] reported that MSCs are short-lived and do not migrate beyond the lungs after intravenous infusion, which was similar to our findings. We observed that despite being short-lived (less than 72 h), the efficacy of S-UCMSCs and UCMSCs, including maintaining visual function and protecting photoreceptors from degeneration, lasted approximately 28 days after transplantation, which was much longer than their presence. It is most likely that intravenous infusion of S-UCMSCs and UCMSCs can continuously regulate the retinal microenvironment through immunomodulatory and neurotrophic effects mediated by paracrine mechanisms, such as decreasing the level of IL-6 and upregulating the level of IL-10 and neurotrophic factors, such as BDNF, CNTF, bFGF and HGF, rather than homing to the retina and differentiating and replacing damaged tissues and cells [1, 56]. However, the validity period of 28 days in this study is limited for the treatment of RP, a chronic progressive disease. Although the differences of the species of objects and research methods are important factors for the results, and our previous clinical study of intravenous transplantation of UCMSCs in RP patients showed that the effectiveness lasted for 12 months, it is still necessary to extend the observation time in the future studies to further explore the duration of effectiveness.

However, the lack of research on the distribution of other tissues of intravenously infused cells in addition to the lung and retina might be a limitation of our study. Since the lung is the organ in which the cells are blocked most heavily compared with other organs and pulmonary complications are more likely to be life-threatening, we focused on the lung in the present study. Nonetheless, the fate and migration pathway of intravenously infused UCMSCs still need to be further explored in subsequent studies to better clarify the therapeutic mechanism and optimize the treatment. In addition, although no cells migrated to the retina in our study, in view of the considerable controversy about cell homing, multiple experimental methods are still needed to further confirm our research results.

In addition, although various approaches for small MSCs, such as filters, centrifuge system, counter flow elution and microfluidic sorting [45, 57, 58], have been developed in addition to nylon filters, there is a lack of unified processes and standards for their preparation, which limits the application of small MSCs. Therefore, based on maintaining biological function, methods to standardize the process and unify cell size for clinical application need to be further studied.

## Conclusions

In summary, compared with UCMSCs, intravenous infusion of S-UCMSCs was safer and could protect visual function and delay retinal degeneration in RCS rats. This study provides a novel strategy to optimize UCMSC transplantation for RP patients, especially when administered repeatedly and the range of subjects expands.

## Supplementary Information

**Additional file 1: Figure S1.** The expression of GFP in UCMSCs. **Figure S2.** Distribution of GFP-positive cells in whole lung fields. **Figure S3.** Representative micrograph of retinal preparation.

## Data Availability

The datasets used and/or analyzed during the current study are available from the corresponding author on reasonable request.

## Abbreviations

S-UCMSCs: Small Umbilical Cord Mesenchymal Stem Cells
ONL: Outer Nuclear Layer
FERG: Flash Electroretinogram
BDNF: Brain-Derived Neurotrophic Bactor
CNTF: Ciliary Neurotrophic Factor
bFGF: Basic Fibroblast Growth Factor
HGF: Hepatocyte Growth Factor
