# Supplementary material for: Intravenous infusion of small umbilical cord mesenchymal stem cells could enhance safety and delay retinal degeneration in RCS rats
Source: BMC Ophthalmol. 2022 Feb 11;22:67. doi: 10.1186/s12886-021-02171-3 (PMC8832832; doi:10.1186/s12886-021-02171-3)
Supplement: Supplementary file 1 — Additional file 1: Figure S1. The expression of GFP in UCMSCs. Figure S2. Distribution of GFP-positive cells in whole lung fields. Figure S3. Representative micrograph of retinal preparation. [file 12886_2021_2171_MOESM1_ESM.docx]

Intravenous infusion of small umbilical cord mesenchymal stem cells could enhance safety and delay retinal degeneration in RCS rats

Qingling Liang^1,2^, Qiyou Li^1,2^, Bangqi Ren^1,2^, Zhengqin Yin^1,2,*^

^1^Southwest Hospital/Southwest Eye Hospital, Third Military Medical University (Army Medical University), Chongqing 400038, China

^2^Key Lab of Visual Damage and Regeneration & Restoration, Chongqing 400038, China

**^*^Corresponding author**

Zheng Qin Yin

Southwest Hospital/Southwest Eye Hospital, Third Military Medical University (Amy Medical University), Chongqing 400038, China

Fax: 86-23-65460711

E-mail: yzhengqin@163.com

Tel: 86-23-68765378

**Supplemental materials**

**Fig. S1.** The expression of GFP in UCMSCs.

**Fig. S2.** Distribution of GFP-positive cells in whole lung fields.

**Fig. S3.** Representative micrograph of retinal preparation.


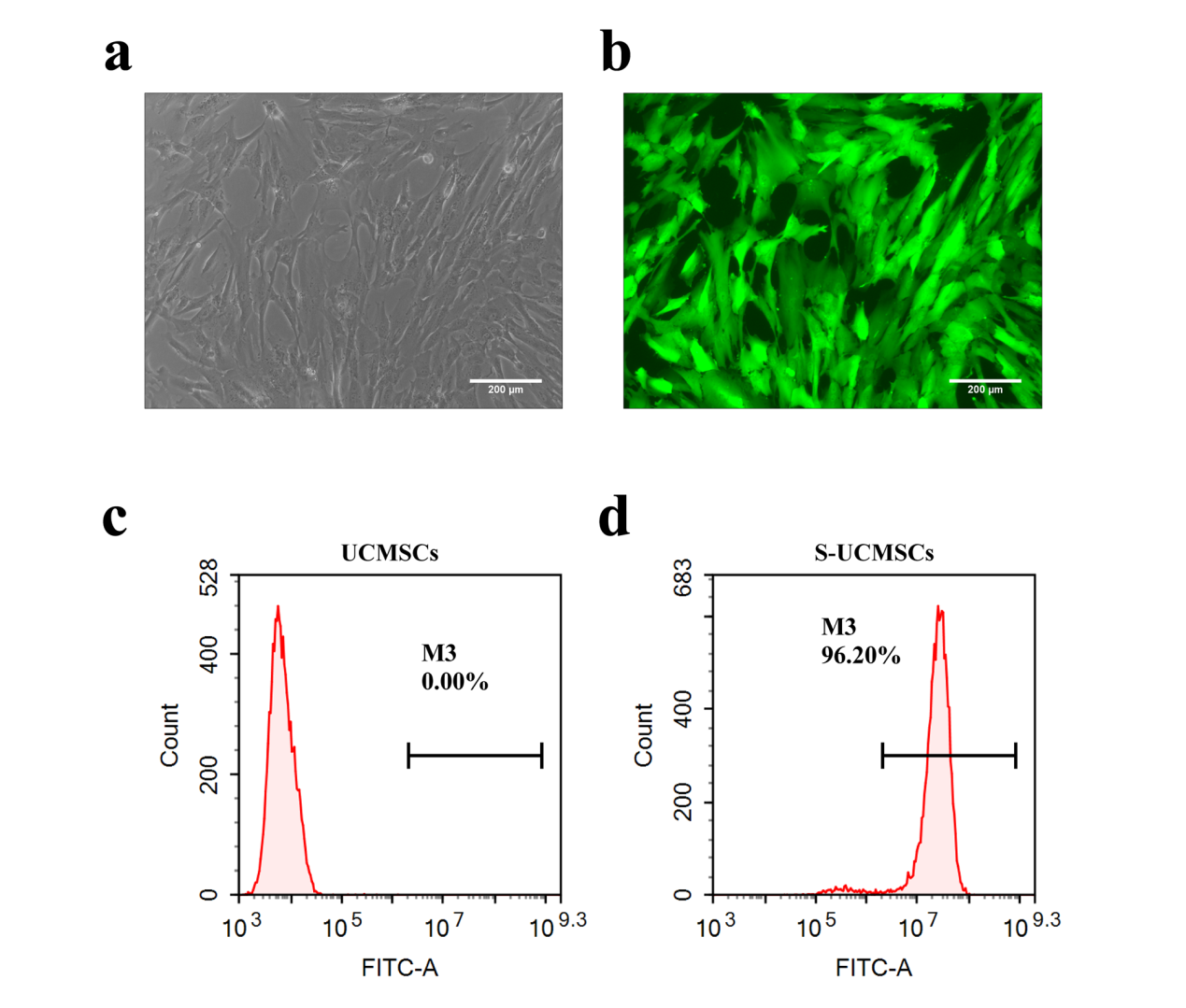


**Fig. S1** The expression of GFP in UCMSCs. Representative micrographs of GFP-negative (A) and GFP-positive UCMSCs (B). (C, D) The GFP fluorescence transfection efficiency of UCMSCs determined by flow cytometry was 96.2%. Scale bar: 100 μm (A and B).


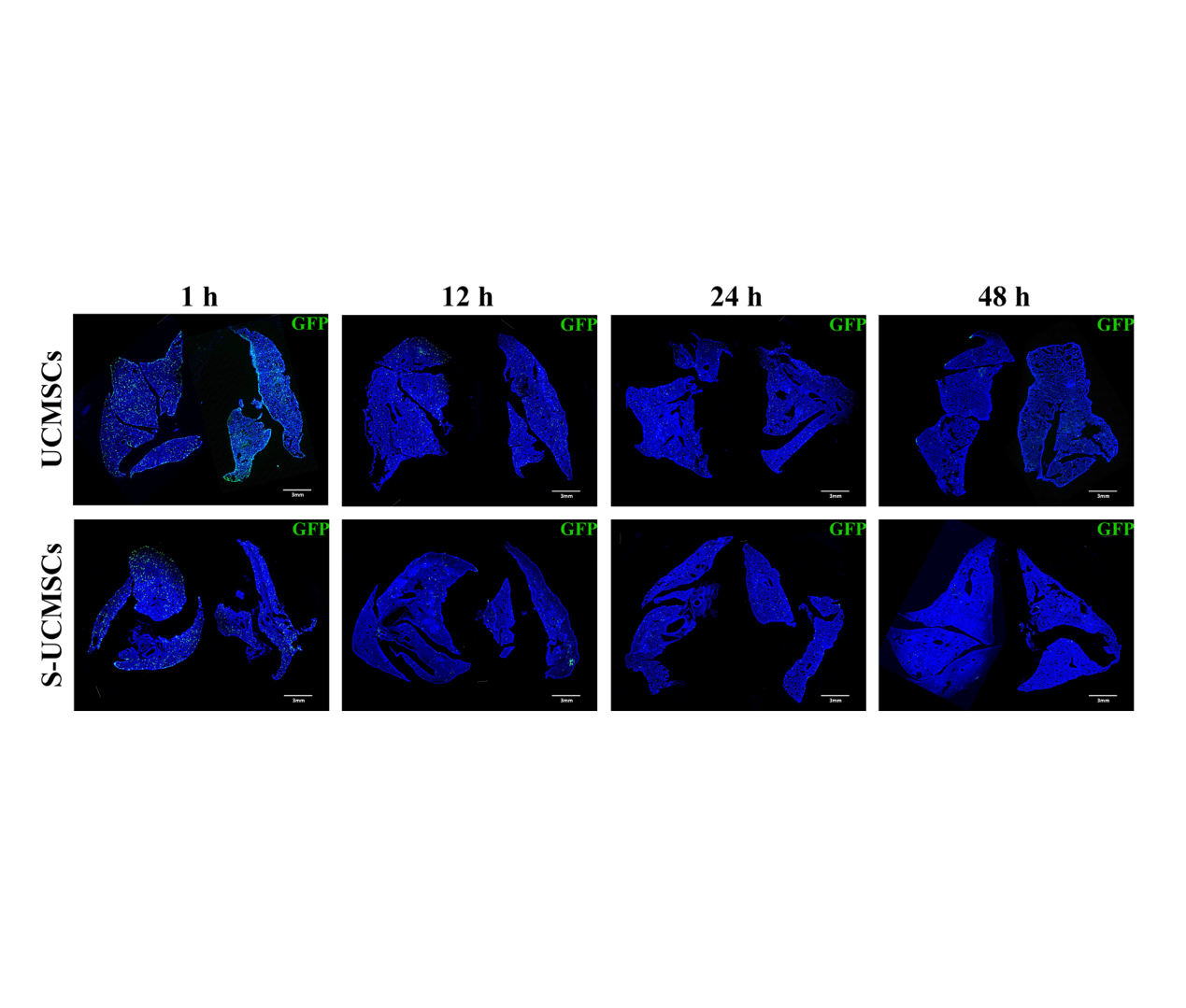


**Fig. S2** Distribution of GFP-positive cells in whole lung fields. The representative micrographs showed that the S-UCMSCs blocked in the lungs were fewer than UCMSCs after transplantation, and the difference was statistically significant at 12 h and 24 h. And the GFP-positive cells in lungs in both groups gradually decreased over time and nearly disappeared at 48 h. Cell nuclei were stained with DAPI. Scale bar: 3 mm.


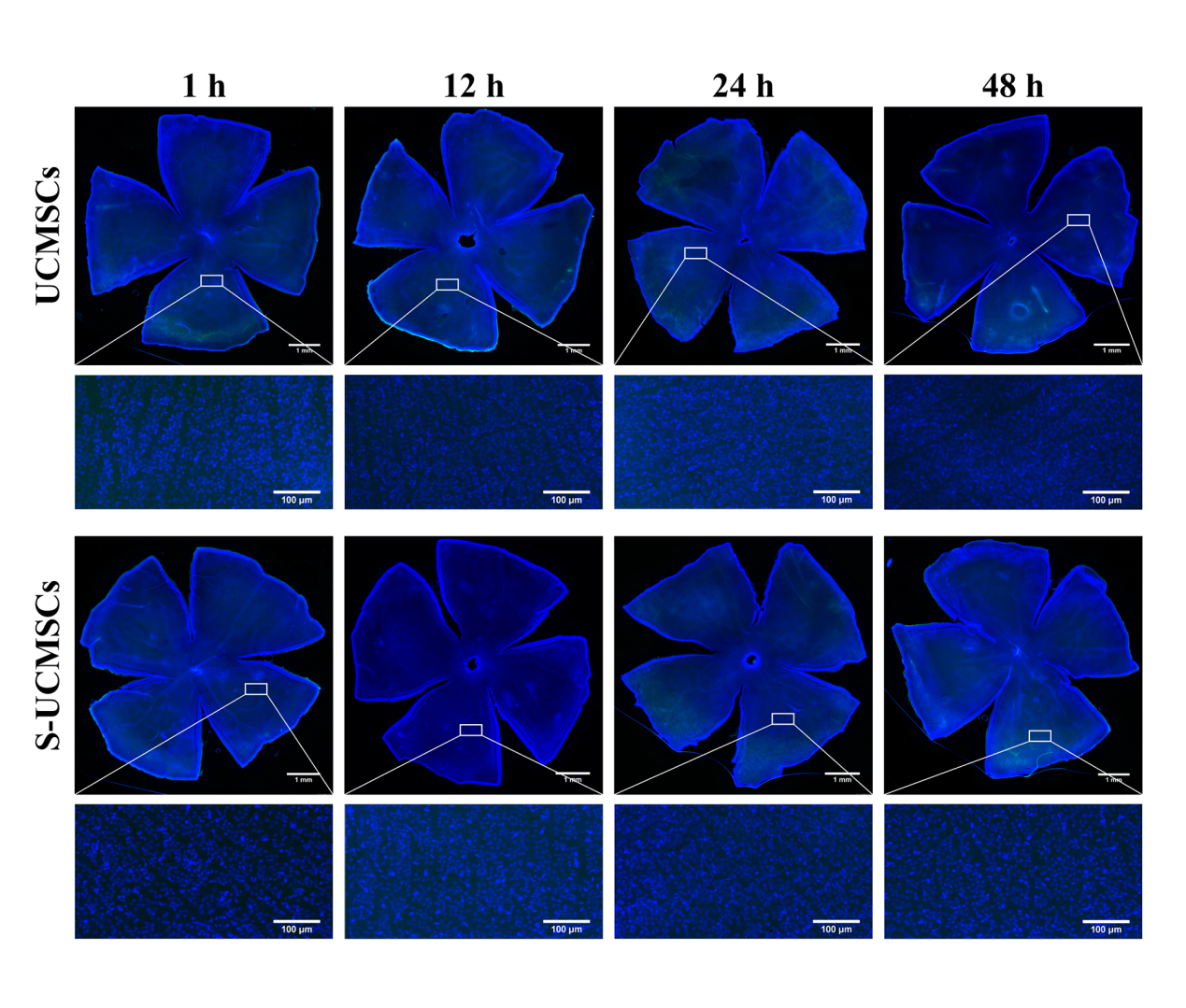


**Fig. S3** Representative micrographs of retinal preparations. The retina preparation and its enlarged areas showed no GFP-positive cell was observed in retina at 1, 12, 24 and 48 h after transplantation. Cell nuclei were stained with DAPI. Scale bars: 1 mm (whole retina preparation) and 100 μm (enlarged micrograph).
